# Protein supplementation changes gut microbial diversity and derived metabolites in subjects with type 2 diabetes

**DOI:** 10.1016/j.isci.2023.107471

**Published:** 2023-07-25

**Authors:** Ilias Attaye, Pierre Bel Lassen, Solia Adriouch, Emilie Steinbach, Rafael Patiño-Navarrete, Mark Davids, Rohia Alili, Flavien Jacques, Sara Benzeguir, Eugeni Belda, Ina Nemet, James T. Anderson, Laure Alexandre-Heymann, Arno Greyling, Etienne Larger, Stanley L. Hazen, Sophie L. van Oppenraaij, Valentina Tremaroli, Katharina Beck, Per-Olof Bergh, Fredrik Bäckhed, Suzan P.M. ten Brincke, Hilde Herrema, Albert K. Groen, Sara-Joan Pinto-Sietsma, Karine Clément, Max Nieuwdorp

**Affiliations:** 1 Department of Internal and Vascular Medicine, Amsterdam University Medical Centers, Location AMC, Amsterdam, the Netherlands; 2 Sorbonne Université, INSERM, Nutrition and Obesities; Systemic Approaches (NutriOmics), Paris, France; 3 Assistance Publique Hôpitaux de Paris, Pitie-Salpêtrière Hospital, Nutrition Department, Paris, France; 4 Department of Cardiovascular & Metabolic Sciences, Lerner Research Institute, Cleveland, OH, USA; 5 Center for Microbiome & Human Health, Cleveland Clinic, Cleveland, OH, USA; 6 Assistance Publique Hôpitaux de Paris, Cochin Hospital, Diabetes department, Paris, France; 7 Unilever Foods Innovation Centre, Wageningen, the Netherlands; 8 Department of Cardiovascular Medicine, Heart, Vascular and Thoracic Institute, Cleveland, OH, USA; 9 Wallenberg Laboratory, Department of Molecular and Clinical Medicine and Sahlgrenska Center for Cardiovascular and Metabolic Research, University of Gothenburg, 413 45 Gothenburg, Sweden; 10 Region Västra Götaland, Sahlgrenska University Hospital, Department of Clinical Physiology, Gothenburg, Sweden

**Keywords:** Dietary supplement, Health sciences, Human metabolism, Microbiome

## Abstract

High-protein diets are promoted for individuals with type 2 diabetes (T2D). However, effects of dietary protein interventions on (gut-derived) metabolites in T2D remains understudied. We therefore performed a multi-center, randomized-controlled, isocaloric protein intervention with 151 participants following either 12-week high-protein (HP; 30Energy %, N = 78) vs. low-protein (LP; 10 Energy%, N = 73) diet. Primary objectives were dietary effects on glycemic control which were determined via glycemic excursions, continuous glucose monitors and HbA1c. Secondary objectives were impact of diet on gut microbiota composition and -derived metabolites which were determined by shotgun-metagenomics and mass spectrometry. Analyses were performed using delta changes adjusting for center, baseline, and kidney function when appropriate.

This study found that a short-term 12-week isocaloric protein modulation does not affect glycemic parameters or weight in metformin-treated T2D. However, the HP diet slightly worsened kidney function, increased alpha-diversity, and production of potentially harmful microbiota-dependent metabolites, which may affect host metabolism upon prolonged exposure.

## Highlights

• Protein supplementation doesn’t change glycemic control in T2D patients
• Gut microbial diversity increases with protein supplementation
• Protein supplementation increases metabolites associated with cardiovascular risk
• Increased metabolites include phenyl-acetylglutamine (PAGln)

## Graphical abstract

**Figure:**
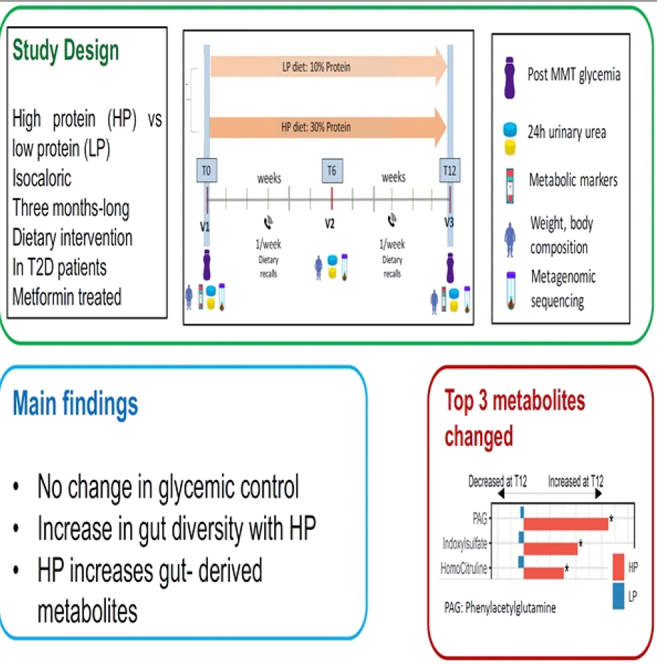

## Introduction

Cardio-metabolic diseases (CMD) represent an umbrella term encompassing, among other, type 2 diabetes (T2D) and cardiovascular diseases.^1^ CMD are currently on the rise globally and are the leading causes of morbidity and mortality.^2^^,^^3^^,^^4^ The pathophysiology of CMD is complex, but one important factor recognized to be involved is insulin resistance, which is a hallmark trait in the onset of T2D and glycemic control in individuals with T2D.^5^ A key element in management of patients with T2D is to provide diet guidance as multiple dietary interventions have shown the potential of diet manipulation to positively affect metabolic health.^6^^,^^7^

Recently, a large focus has been put on modulation of dietary protein levels,^8^^,^^9^^,^^10^ due to its associations with cardiometabolic risks.^11^ Protein, in particular animal protein consumption, has been linked with increased risk of T2D development in observational prospective and meta-analysis studies.^12^^,^^13^^,^^14^^,^^15^ On the other hand, several studies have reported the potential benefit of high protein (HP) diets by inducing weight loss and associated improvement of metabolic parameters.^16^^,^^17^ However, in intervention studies with modulation of protein intake the effects on metabolic and glycemic markers are more ambiguous. In some reports, HP dietary intake was linked with improvement of glycemic control whereas other studies showed a detrimental effect.^18^^,^^19^ Other studies reported little to no effects of increased protein intake on metabolic parameters in individuals with T2D.^20^ Importantly, most HP dietary interventions include a caloric restriction and, therefore, weight loss is an important confounder making it difficult to truly distinguish the effect of protein modulation from the metabolic effect of weight loss.^6^^,^^21^^,^^22^^,^^23^

The pathophysiology behind these conflicting results remains complex. However, in recent years, it has become clear that the gut microbiota, an endocrine organ that encompasses trillions of microbes, plays a key role in metabolic health.^24^^,^^25^^,^^26^^,^^27^ The gut microbiota itself is dynamic in its composition and function and can be modulated via several routes (e.g., medication use and host ethnicity lifestyle including diet).^28^^,^^29^^,^^30^ One of the main mechanisms by which the gut microbiota can influence host health is through the generation of microbial derived metabolites, which can be produced from the host diet.^31^^,^^32^^,^^33^

As such rodent and clinical studies have shown the potential of fiber intake to promote the production of beneficial short-chain fatty acids (SCFA) that can improve insulin sensitivity and positively affect multiple metabolic pathways.^34^^,^^35^^,^^36^ Conversely, several detrimental microbial metabolites can be formed from dietary (animal) protein. These metabolites include, among others trimethylamine (TMA), which is generated by bacteria from dietary carnitine, choline, and betaine. In the liver, TMA is converted to trimethylamine-N-oxide (TMAO), which has been extensively linked to poor cardiovascular outcome.^37^^,^^38^ Other important metabolites are phenylacetylglutamine (PAG) and imidazole propionate (ImP). PAG is formed from the essential amino acid phenylalanine and has been linked to increased platelet reactivity and higher prevalence of CMD.^39^ ImP is produced by bacteria using the essential amino acid histidine as a substrate and has been linked to increased insulin resistance, T2D status, and reduced effect of the anti-diabetic drug metformin.^40^^,^^41^^,^^42^

Some studies have investigated the effects of an HP diet on the gut microbiota composition;^43^^,^^44^ however, most data describing the interaction between dietary protein intake and metabolite production come from animal or observational studies.^45^ Thus, evaluating the effect of HP consumption on microbial metabolites and the interaction with host metabolism merits consideration.

We therefore performed a 12-week randomized, multi-center, isocaloric dietary intervention comparing high protein (HP; protein: 30% of energy intak[ (En%]) vs. low protein (LP; protein: 10% En%) intake in a multi-ethnic population of individuals with T2D on stable metformin. Our primary objective was to investigate the effects of this isocaloric protein modulation on glycemic control and insulin resistance markers in individuals with T2D. Secondary objectives were to analyze the impact of an increase in protein consumption on gut microbiota composition and functional metabolic output as monitored by plasma levels of microbiota-derived metabolites.

## Results

### Baseline characteristics

**Figure 1:**
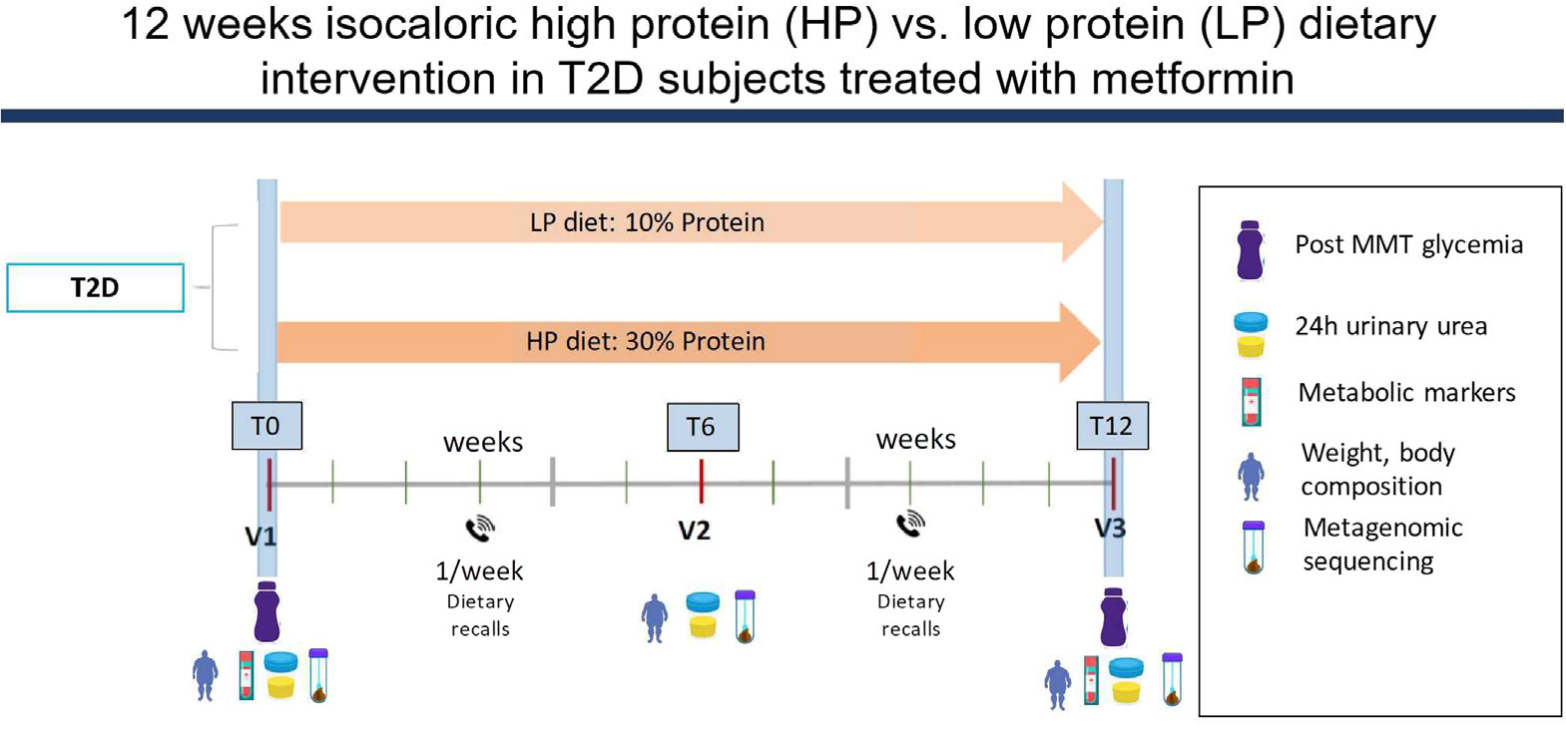
Study design MICRODIET trial Subjects were randomized to follow either a high protein (HP) or low protein (LP) diet for 12 weeks. Study visits were performed at week 0 (baseline), week 6, and week 12 (end of intervention). A mixed-meal test (MMT) was performed at week 0 and week 12 and plasma for metabolomics was also obtained. Dietary adherence was observed through weekly contact with a dietician and the use of weekly food diaries. Before each study visit subjects collected 24-h urine and as well as 24-h fresh feces.

**Figure 2:**
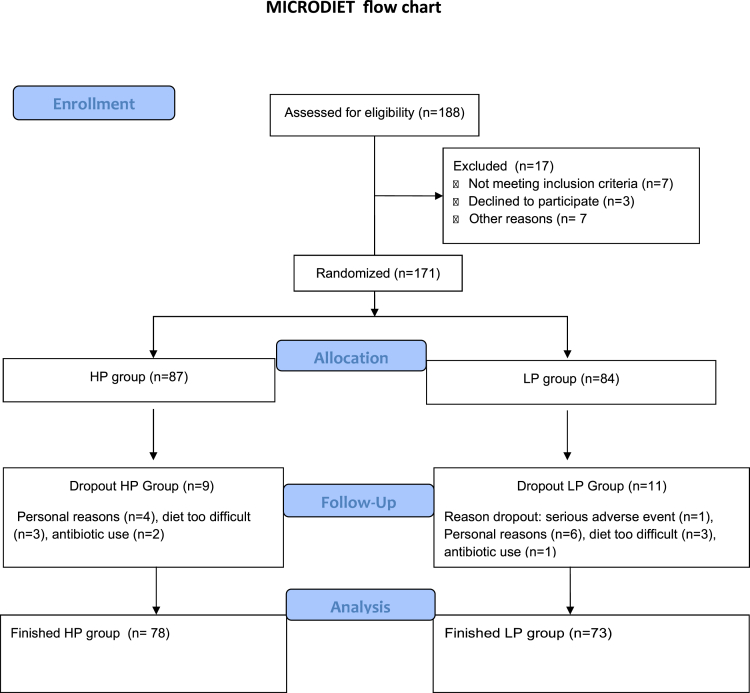
CONSORT flowchart inclusion MIRCODIET Trial.

A total of 171 subjects were randomized to either the HP or LP group. The study design is displayed in Figure 1. Out of these subjects, 151 (78 HP and 73 LP diet) finalized the clinical trial and were included in the analyses. Most common reasons for dropout were antibiotic usage during the trial (N = 3), failure to adhere to the diet (N = 6), or personal problems (N = 6) (Figure 2 for CONSORT flow chart). Baseline characteristics are shown in Table 1. At baseline there were no statistically significant differences in biochemical or anthropometric parameters. Subjects had a mean age of 58.2 ± 7.7 years in the HP group and 59.1.1 ± 7.0 years in the LP group. The groups mainly consisted of women, with 56.4% in the HP group and 58.9% in the LP group. All subjects were on stable metformin therapy and a subset also used other diabetes lowering drugs, such as sulfonylureas. However, these subjects were equally distributed among the HP and LP groups. Of note, there were also no statistically significant differences at baseline when subjects were stratified according to center (Table S1).

**Table 1:** Baseline characteristics

Total	HP	LP	p
n	78	73	
Age (years)	58.22 (7.72)	59.06 (7.03)	0.488
Sex = M (%)	34 (43.6)	30 (41.1)	0.885
Ethnicity = Caucasian (%)	40 (51.3)	36 (49.3)	0.937
BMI (kg/m2) (mean (SD)	30.74 (4.17)	31.46 (4.60)	0.314
Fat (%)	31.73 (11.48)	34.01 (11.23)	0.228
Free Fat Mass (%)	60.88 (17.80)	59.57 (13.55)	0.622
Creatinine (mmol/L) (mean (SD))	71.00 (19.59)	67.97 (17.13)	0.319
eGFR_MDRD (mL/min) (mean (SD))	106.03 (30.61)	109.78 (33.16)	0.474
Fasting glucose (mmol/L) (mean (SD)	8.45 (2.33)	8.03 (1.74)	0.206
Fasting insulin (pmol/L) (mean (SD)	71.47 (47.68)	71.89 (36.62)	0.953
HbA1c (mmol/mol) (mean (SD)	57.28 (11.97)	54.35 (8.37)	0.088
LDL (mmol/L) (mean (SD)	2.49 (1.10)	2.44 (0.84)	0.726
HDL (mmol/L) (mean (SD)	1.30 (0.34)	1.36 (0.38)	0.371
Triglycerides (mmol/L) (mean (SD)	1.51 (1.19)	1.51 (0.85)	0.99
CRP (mg/L) (mean (SD)	2.61 (2.08)	4.30 (7.49)	0.106
REE (kcal) (mean (SD)	1566.50 (353.16)	1561.03 (283.03)	0.927
SU derivatives = y (%)	19 (24.4)	26 (35.6)	0.182
GLP1 agonists = y (%)	6 (7.7)	7 (9.6)	0.901
DPP4 inhibitors = y (%)	16 (20.5)	11 (15.1)	0.509
Statin = y (%)	47 (60.3)	39 (53.4)	0.495
Anti-hypertensive drug = y (%)	44 (56.4)	36 (49.3)	0.478

Data are expressed as mean ± SD or %. Student t-test was used for comparison between the groups.

eGFR, estimated glomerular filtration rate; REE, Resting Energy Expenditure; SU derivatives, sulfonylurea derivatives; GLP-1 agonist, glucagon-like-peptide 1 agonist; DDP-4 inhibitors, Dipeptidyl peptidase-4 inhibitors.

### Compliance to the diet

**Figure 3:**
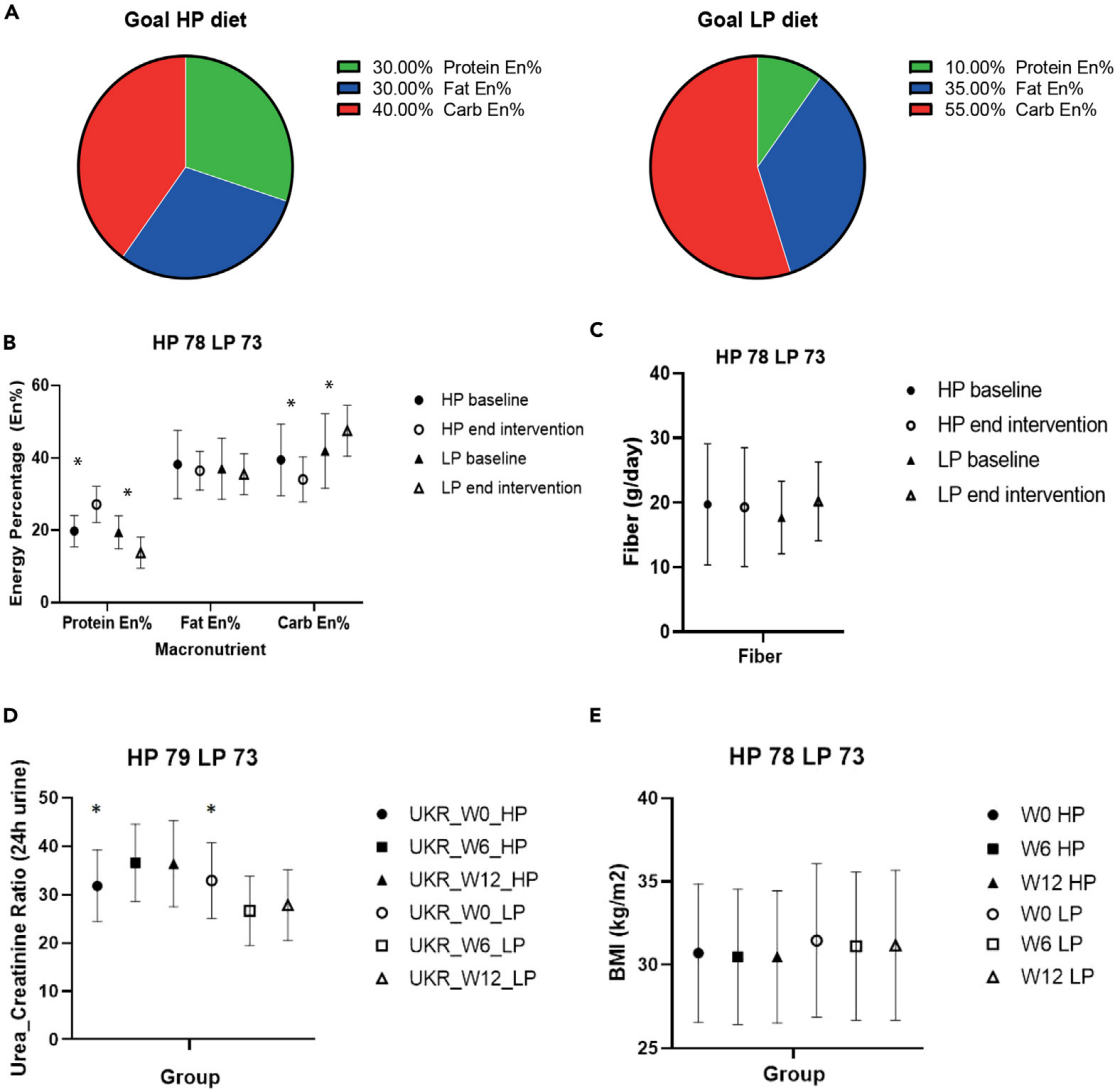
Overview dietary adherence and BMI throughout the study period (A) Shows the target diet composition in the high protein (HP) and low protein (LP) group. (B) Self-reported macronutrient consumption at baseline and end of the study period. Significant differences in protein energy percentage (En%) and carbohydrate intake between baseline and end of intervention period in HP and LP group. The effect of the intervention (HP vs. LP) on changes from baseline (delta between week 12 and week 0) was analyzed in a linear regression model adjusting for baseline values and center. (C) Fiber intake between HP and LP group throughout the intervention (ns). (D) 24-h urine urea/creatinine ratios. In the HP group statistical significant increase between week 0 and week 6 and week 0 and week 1, no statistical significance between week 6–week 12. In the LP group statistical significant decrease between week 0 and week 6 and week 0 and week 12, no statistical significance between week 6–week 12. Data were analyzed using a linear-mixed effects model with post-hoc Dunn’s correction. (E) BMI (body-mass index) between HP and LP group throughout the intervention (ns). All data are presented as mean ± SD. A p < 0.05 was considered statistically significant (indicated with an ∗).

The self-reported compliance with dietary recalls was good in order to match the target diet (Figures 3A and 3B). Baseline protein intake did not differ between the two groups with 19.7 ± 4.3 En% in the HP group and 19.4 ± 4.5 En% in the LP group. At the end of the intervention, protein intake reached 27.2 ± 5.0 En% in the HP vs. 13.8 ± 4.3 En% in the LP group (p < 0.001). The main other excepted change in macronutrient composition of the diet was carbohydrate intake which decreased from 39.5 ± 9.8 En% to 34.0 ± 6.2 En% in the HP group and increased from 41.9 ± 10.3 to 47.5 ± 7.1 in the LP group (p < 0.01). Fat and fiber intake remained stable throughout the study (Figures 3B and 3C). Importantly, the increase in protein was mainly due to an increase in animal protein consumption in the HP group. Saturated fat intake was slightly higher in HP vs. LP group. Plant protein intake did not differ during the intervention between the two groups. The increase in carbohydrates in the LP group was mainly related with higher starch intake with no difference in sugar intake between the two groups (Table 2).

**Table 2:** Detailed dietary follow-up data during the 12-week intervention collected from 24 h recall diaries

	Total	HP	LP	p
n	150	78	72	
Kcal(mean(SD))	1456.5(417.0)	1551.4(432.4)	1395.3(386.4)	0.02
Total protein(gr) (mean(SD))	74.2(37.2)	63.9(28.0)	47.9(15.2)	<0.01
Animal protein(gr) (mean(SD))	45.7(30.0)	98.5(34.8)	26.2(17.2)	<0.01
Plant protein(gr) (mean(SD))	19.1(7.7)	19.0(9.4)	19.1(5.4)	0.93
Carbohydrates (gr) (mean(SD))	148.4(49.6)	135.8(41.1)	162.0(54.6)	<0.01
Fiber (gr) (mean(SD))	18.78(7.8)	19.29(9.2)	20.18(6.1)	0.25
Sugar(gr) (mean(SD))	33.4(28.4)	30.5(24.0)	36.4(32.3)	0.27
Fat (gr) (mean(SD))	60.8(21.2)	67.7(21.8)	56.6(19.9)	0.02
Saturated fat (gr) (mean(SD))	20.6(8.6)	22.9(8.4)	18.1(8.2)	<0.01

The 24 h recall data were collected on 3 randomized days per week during the intervention. Data are expressed as mean ± SD. Student t-test was used for comparison between the groups. A p < 0.05 was considered statistically significant.

Urea/creatinine ratio was not different between the two groups at baseline, but increased significantly in the HP group from 31.8 ± 7.4 at baseline to 36.6 ± 8.0 in week 6 (p = 0.03) and 36.4 ± 8.9 mmol/24-h at week 12 (p < 0.01) compared to baseline. In the LP group, urea/creatinine ratio decreased significantly from 32.9 ± 7.8 at baseline to 26.7 ± 7.3 in week 6 (p < 0.01) and 27.8 ± 7.2 mmol/24 h at week 12 (p < 0.01). There were no differences in urea/creatinine ratios between week 6 and week 12 when comparing the LP and HP groups. (Figure 3D). Moreover, BMI remained stable throughout the intervention (Figure 3E).

### An isocaloric high vs. low protein dietary intervention for 12 weeks does not affect glucose metabolism in metformin treated type 2 diabetes patients

**Figure 4:**
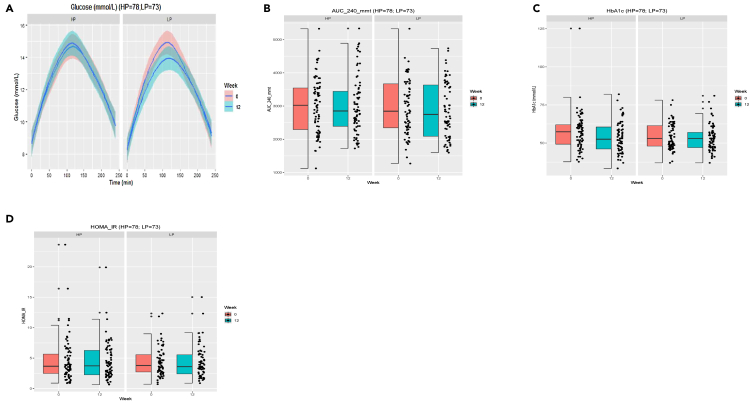
Effect of dietary intervention on glycemic parameters (A) Glucose excursions following a mixed-meal test (MMT) at week 0 and week 12 for 240 min. Data are represented as mean ± SD, no significant changes between the HP or LP group. (B) Area under the curve (AUC) of MMT test performed at baseline and week 12 (ns). (C) No significant effect of dietary intervention on HbA1c between week 0 and week 12. (D) No significant changes in Homeostatic Model Assessment for Insulin Resistance (HOMA-IR) due to the dietary intervention at baseline and week 12. The effect of the intervention (HP vs. LP) on changes from baseline (delta between week 12 and week 0) was analyzed in a linear regression model adjusting for baseline values and center.

We found that neither an HP or LP diet, when administered for 12 weeks, significantly affected glucose metabolism as area under the curve (AUC) after a mixed-meal test (MMT), HbA1c and HOMA-IR did not differ between baseline and end of the intervention (Figures 4A–4D). Moreover, post-prandial insulin response, c-peptide levels as well as results from the continuous glucose monitor performed throughout the study showed large interindividual differences and, therefore, no significant effect (p > 0.1 for all outcomes) of the dietary intervention, even when excluding extreme outliers (Figures S1 and S2).

Analyses were also performed after stratification to baseline gut microbiota diversity (high vs. low Shannon diversity), which did not affect the primary outcome (Figure S3).

### A 12-week isocaloric high vs. low protein dietary intervention does not affect BMI, body composition or biochemical parameters with the exception of renal function

As previous studies have reported both beneficial (increased satiety and weight loss), as well as detrimental (renal damage) effects of a HP diet, we next determined the effects of an HP vs. LP diet on body weight, body composition, and biochemical parameters (Figure 3E; Table 3). This study did not find significant weight changes or body composition changes following the isocaloric intervention. Moreover, lipid levels, inflammation markers, and energy expenditure were not affected. However, estimated renal function decreased in the HP group by 1.67 ± 15.3 mL/min/1.73m2 and improved in the LP group 3.0 ± 12.98 mL/min/1.73m2 (p = 0.03).

**Table 3:** Effect of 12-week dietary protein intervention on anthropometric and biochemical values

Total	HP	LP	Delta HP	Delta LP	p
n	78	73	78	73	
BMI (kg/m2) (mean (SD)	30.55 (3.96)	31.17 (4.50)	−0.29 (0.84)	−0.38 (0.75)	0.63
Fat (%)	31.83 (10.55)	33.42 (10.82)	−0.15 (5.14)	−0.99 (3.67)	0.43
Free Fat Mass (%)	60.77 (16.38)	60.06 (14.08)	−0.66 (5.36)	0.58 (4.28)	0.24
Creatinine (mmol/L) (mean (SD))	73.15 (23.13)	66.73 (16.77)	1.82 (10.13)	−1.57 (7.09)	0.02
eGFR_MDRD (mL/min) (mean (SD))	104.08 (30.60)	110.90 (29.76)	−1.67 (15.32)	3.00 (12.98)	0.03
Fasting glucose (mmol/L) (mean (SD)	8.26 (2.27)	7.83 (1.74)	−0.23 (1.64)	−0.16 (1.33)	0.76
Fasting insulin (pmol/L) (mean (SD)	72.31 (47.19)	73.57 (38.16)	0.83 (38.63)	1.26 (24.97)	0.90
HbA1c (mmol/mol) (mean (SD)	53.96 (10.00)	53.16 (8.67)	−3.27 (9.15)	−1.09 (6.35)	0.42
LDL (mmol/L) (mean (SD)	2.36 (1.09)	2.35 (1.19)	−0.13 (0.66)	−0.09 (0.94)	0.76
HDL (mmol/L) (mean (SD)	1.33 (0.39)	1.32 (0.42)	0.02 (0.16)	−0.02 (0.15)	0.06
Triglycerides (mmol/L) (mean (SD)	1.35 (0.88)	1.51 (0.90)	−0.15 (0.71)	0.01 (0.55)	0.07
CRP (mg/L) (mean (SD)	3.79 (7.09)	3.27 (4.39)	1.18 (7.37)	−1.16 (7.81)	0.44

Data are expressed as mean ± SD or %. The effect of the intervention (HP vs. LP) on changes from baseline (delta between Week 12 and Week 0) was analyzed in a linear regression model adjusting for baseline values and center. eGFR= estimated glomerular filtration rate.

### Dietary protein intake affects gut microbiota composition and plasma metabolite profile

**Figure 5:**
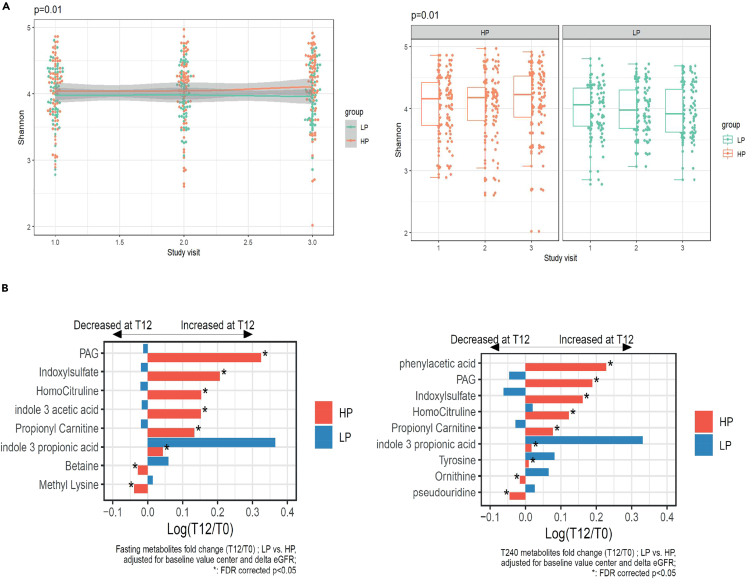
Effect of dietary intervention an alpha diversity (A) Effect of dietary intervention an alpha diversity (Shannon index). A high protein diet increased Shannon index (p = 0.01). (B) Fasting plasma metabolite levels and post-prandial metabolite levels 240 minutes after a mixed meal test. A high protein diet induces several changes in plasma metabolome both fasting (left panel) and 240 min post MMT (right panel). Metabolite fold changes were analyzed after log transformation in a linear model correcting for baseline value, center and delta estimated glomerular filtration rate (eGFR) . All analyses were corrected for false discovery rate (FDR). PAG: Phenylacetylglutamine.

We next determined the effects of the dietary intervention on gut microbiota composition and diversity using metagenomic approaches (Figure 5A). This 12-week HP vs. LP intervention did elicit changes in the gut microbiota composition albeit with a modest effect; the explained variance of the beta-diversity was 0.146% (p < 0.001) (Figure S4). Moreover, alpha diversity, increased in the HP group by 2.6% and decreased in the LP group by 0.2% (HP vs. LP p = 0.01) (Figure 5A). Interestingly, the effects of the protein intervention on gut microbiota composition were not driven by large changes in individual taxa (Figure S5). Moreover, the respective diets did not induce significant functional changes in the gut microbiota (Figure S6).

As gut microbiota composition is related to microbiota-derived metabolite production, we next measured if the protein interventions elicited changes in the measured plasma metabolite profile (Figure 5B). We therefore determined fasting and postprandial (t = 240 min during MMT) plasma levels of several (protein-derived) metabolites. Interestingly, a majority of the observed metabolite changes were driven by increases of the metabolite levels in the HP group. The main metabolites that increased in the HP group were PAG (log fold change: 0.32 i.e., 38% increase); indoxyl sulfate (log fold change: 0.21 i.e., 23% increase), indole-3-acetic acid (log fold change: 0.15 i.e., 16% increase), homocitruline (log fold change: 0.15 i.e., 16% increase), and propionyl carnitine acid (log fold change: 0.13 i.e., 14% increase) in the fasting metabolites. In the postprandial samples phenylacetic acid, PAG, indoxyl sulfate, homocitruline, and propionyl carnitine were significantly affected by the dietary intervention. In the LP group, changes were more limited but we found an increase in indole 3 propionic acid (log fold change: 0.43 i.e., 44% increase). All previously described shifts in metabolites were statistically significant after correction for baseline value, center, and estimated glomerular filtration rate (eGFR). No changes on ImP levels were induced by the protein modulation.

**Figure 6:**
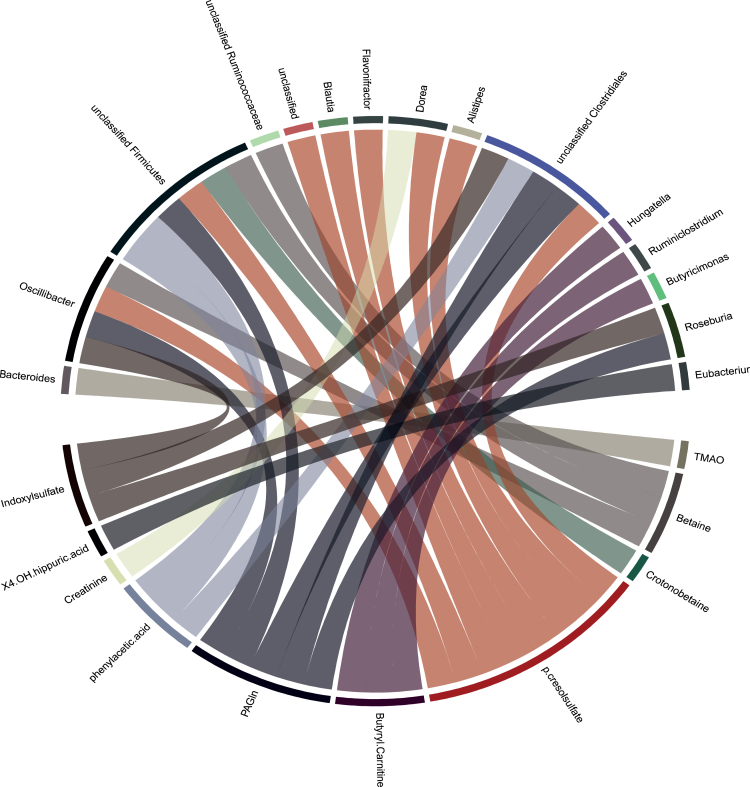
Associations between individual taxa and plasma metabolite levels Displayed are only significant associations using linear mixed effect models and false discovery rate (FDR) correction.

To determine the relationship between taxa- and plasma metabolite levels, we tested associations with linear mixed effect models and found several relationships between individual taxa and an increase in the associated metabolite (Figure 6). The results show cross-sectional associations displaying the relationship between individual taxa and associated serum metabolites. However, when taking the dietary intervention into account, only four taxa-metabolite relationships remained significant, and most associations were therefore driven by the dietary intervention. The HP diet increased the *Dorea* sp*.CAG:105*, which was correlated with an increase in *p*-cresol sulfate; Firmicutes *bacterium CAG:102* and *Oscilibacter* sp (both correlated with an increase in PAGln) and *Roseburia* sp (which correlated with an increase in Indoxyl sulfate). The LP diet was associated with decreased levels of aforementioned species and metabolites, indicating again a diet-driven response (Figure S7).

## Discussion

In this 12-week randomized HP vs. LP dietary intervention without caloric restriction in individuals with T2D treated with metformin, we did not observe any significant effects of protein modulation on post prandial glycemic excursions, HbA1c, and other metabolic parameters. However, protein supplementation induced a small increase in microbial diversity and significant changes in gut microbial-derived metabolomic profiles.

We found no effect of a short-term HP vs. LP dietary intervention on glycemic markers despite detailed phenotyping using post-prandial glycemic excursion after a MMT, HbA1c, HOMA-IR, and real-world data with continuous glucose monitoring. Importantly, long-term observational studies have shown an association between increased animal protein intake and T2D incidence, whereas plant protein consumption had a neutral effect.^12^^,^^13^^,^^14^^,^^15^ These results are not contradicted by our study performed in individuals with installed T2D but should not be extrapolated to individuals without T2D for whom protein intake has been associated with increased insulin secretion and hepatic production of glucose, which ultimately may lead to increased insulin resistance and T2D development.^46^ On the other hand, in individuals with T2D, low-calorie HP dietary interventions reported improved metabolic markers^16^^,^^17^ contrary to our results. However, in these studies, the effects of HP diet may have been confounded by the caloric-restriction induced weight loss, which was more important in HP diets and is a confounding factor when studying metabolic parameters. Here, we carefully controlled this potential confounder by providing isocaloric diet in both HP and LP groups. Consequently, the main change was the modulation of macronutrients as shown by the little observed weight loss (approximately 1 kg), which was not different between the two groups. Therefore, this study allows to interpret solely the effect of protein modulation without the bias of differences in weight loss between the groups.

Renal function was significantly changed by the protein intervention with a decrease in the HP group opposed to an improvement in the LP group. The effect was rather small with little clinical relevance, but this result questions what may be the outcomes of longer-term HP diets on renal function. Previous meta-analysis showed that indeed, an LP diet may improve renal function in T2D, without effecting glycemic parameters.^47^ Whether an HP diet significantly and clinically worsens renal functions remains a topic of discussion^48^

In this controlled setting, the HP vs. LP intervention induced an increase in gut microbiota diversity, due to the HP group. Previous studies in individuals without T2D have reported that a HP, caloric-restricted dietary intervention was associated with an increase in microbiota diversity sometimes reaching 30% increase.^49^^,^^50^ Our result suggests that HP diet per se (e.g., without calorie restriction) could be associated with increased alpha diversity albeit with a low magnitude. Indeed, the observed increase in diversity is moderate (2.4% increase in Shannon index) and probably not sufficient to restore microbiota richness in subjects with severe dysbiosis which has been linked with metabolic diseases.^51^ This suggests that to significantly and more dramatically increase microbial diversity, combining caloric restriction with macronutrient changes might be more efficient.

Furthermore, this study found that protein modulation did result in changes in plasma metabolite profile, mainly driven by the HP group. Of note, these changes were seen without large effects on gut microbiota composition or function as evaluated via metagenomics analysis. One possible explanation for this, is the potential of diet to induce post-translational changes,^52^ i.e., changes that can be more pronounced in the transcriptome which is undetectable via metagenomics.

The main metabolites monitored that increased due to the HP diet were PAG and indoxyl sulfate, both of which have been clinically and mechanistically associated with cardiovascular risk.^39^^,^^53^^,^^54^ PAG is a gut-derived metabolite produced from the essential amino acid phenylalanine. Increased PAG levels have been associated with cardiovascular disease and increased thrombosis potential.^39^ Indoxyl sulfate is a gut-derived uremic toxin which has been linked to chronic kidney disease and cardiovascular disease, possibly via increased inflammation, endothelial dysfunction, and higher levels of cardiac fibrosis.^53^

In the LP group the main metabolite change was a significant increase in indole-3-propionic acid. This gut microbiota generated metabolite of the essential amino acid tryptophan may be a protective factor against atherosclerosis by promoting reverse cholesterol transport and is down-regulated in patients with atherosclerotic cardiovascular disease.^55^ Moreover, indole-3-propionic acids has been clinically and mechanistically been linked to diabetes and other metabolic disorders.^56^ The protein content of the diet had a neutral impact on ImP, a metabolite of the essential amino acid histidine. Although this result may be surprising, it confirms the absence of link between histidine consumption and ImP levels previously reported in an observational study from the European MetaCardis population.^57^

This further supports the hypothesis that dietary protein may be metabolized in the small intestine but that other macronutrients, e.g., dietary fiber, can modulate the gut microbiota structure and function thus affecting enzyme activities and metabolite production where substrates originate from host or microbial proteins.^40^

On the other hand, we did not observe significant metagenomic functional changes with the intervention whereas metabolites change significantly with a relatively important effect size. This suggests that for these metabolites the most modification of the gut environment with the protein modulation was sufficient to change their production without changes in abundance of metagenomic functions. It is also possible that duration of the intervention was not sufficient to induce significance metagenomic functional shifts and that a longer intervention would have resulted in both metagenomic functional changes and metabolites changes.

Since diet induced small, but significant, changes in gut microbiota diversity and also led to a change^58^ in plasma metabolite profile, we next determined associations between microbial taxa and plasma metabolites. This study identified several associations between taxa and metabolites, such as *Oscillibacter* and indoxyl sulfate levels, which has been previously reported.^58^ However, when specifying to the intervention in this study, only four associations remained. The HP diet increased the *Dorea* sp*.CAG:105*, which was correlated with an increase in p. cresolsulfate; *Firmicutes bacterium CAG:102*, *Oscilibacter* sp (both correlated with an increase in PAGln), and *Roseburia* sp (which correlated with an increase in Indoxyl sulfate). The LP diet decreased aforementioned species and metabolites. More research is warranted in these specific taxa-metabolite relationships, as these can serve as potential targets of specific dietary interventions.

In conclusion, this multi-center, 12-week, randomized-controlled isocaloric, dietary protein intervention in individuals with T2D subjects on metformin treatment showed that a short-term protein modulation does not affect glycemic parameters, but an HP diet results in a small increase in serum creatinine. Moreover, the HP diet vs. the LP diet leads to changes in gut microbiota composition and production of (gut-derived) metabolites which are themselves known to be associated with cardiovascular risk. Furthermore, this study identified several taxa-metabolite associations that were diet driven. More research is needed with longer duration and study population in order to investigate and validate potential causal effects of these findings in CMD.

### Limitations of the study

This study has limitations and strengths. A first limitation is the relatively short duration of the study and exposition to the dietary modification. Indeed, it is possible that with a more prolonged exposure, the dietary intervention may have had a significant effect on metabolic outcomes. Another limitation is that our sample size may have not been large enough to detect smaller metabolic changes. However, our power analysis did show sufficient power for the primary outcome. Moreover, the population studied was heterogeneous and used several antidiabetic drugs, which could have influenced gut microbiota composition and function as shown in previous metagenomic analysis.^59^ Although half of the diet was directly provided to the patients, we did not control all of the dietary and beverage intake of the participants during the study, as this was not feasible for such a relatively large group. Another limitation is the fact that, by design, protein was not the only macronutrient modified by the dietary intervention study, as carbohydrates were also significantly changed in order maintain an isocaloric diet. However, we can note that the main difference in terms of dietary intake between the 2 groups is the protein content which is double the amount in HP vs. LP when the difference in carbohydrates or fat intake between the two groups is much less pronounced. Moreover, fiber intake was kept constant throughout the study to avoid confounding effects. Moreover, this study only investigated post-prandial metabolite changes after 240 min at baseline and after a 12 weeks intervention. However, this study did identify several metabolites that had altered post-prandial levels after 12 weeks of protein intervention compared to baseline. However, studies are needed investigating longer post-prandial timepoints to ensure that these changes are not transient, but can, with prolonged duration of intervention, affect host metabolism.

Strengths of this study included the detailed phenotyping on both metabolic, as well as microbial level. Moreover, this study investigated a heterogeneous, real world, multi-ethnic population which increases the generalizability of the findings. The results of this study add to the ongoing debate whether animal protein intake can be associated with negative metabolic outcomes.^11^ This is of importance as this is one of the first studies investigating the effect of protein modulation in an isocaloric fashion taking also the effect on gut microbiota composition and gut-derived metabolites into account. Lastly, this study was not confounded by weight changes, as the majority of dietary intervention studies are which allows a proper interpretation of macronutrient modulation.

## STAR★Methods

### Key resources table

**Table:** 

REAGENT or RESOURCE	SOURCE	IDENTIFIER
**Biological samples**		

Human plasma metabolomics data	This study (PI Prof. Dr. M. Nieuwdorp)	N/A
Human fecal metagenomics data	This study (PI Prof. Dr. M. Nieuwdorp)	N/A

**Chemicals, peptides, and recombinant proteins**		

Histidine-D5,15N3	Cambridge Isotope Lab. Inc, Cambridge, UK	(Koh et al. 2018)^40^
imidazole propionate-13C3	Astra Zeneca, Cambridge, UK	(Koh et al. 2018)^40^
urocanate-13C3	Astra Zeneca, Cambridge, UK	(Koh et al. 2018)^40^

**Critical commercial assays**		

DNA extraction kit	QIAamp DNA Mini kit	N/A

**Software and algorithms**		

open software program R version 4.2.1	(R Core Team (2022). R: A Language and Environment for Statistical Computing. R Foundation for Statistical Computing, Vienna, Austria. URL Https://Www.R-Project.Org/., ND)	https://www.R-project.org/
MEDUSA pipeline	N/A	(Karlsson et al. 2014)^72^

**Other**		

Freestyle Libre Glucose Monitoring System	ABBOTT DIABETES CARE IN FRANCE Abbott France S.A.S. Abbott Diabetes Care 94528 Rungis Cedex France	version 1.0
Nutridrink Compact Protein	Nutricia Advanced Medical Nutrition, Amsterdam, the Netherlands	Strawberry compact protein
Food diary log “Mijn Eetmeter”	Stichting Voedingscentrum Nederland	N/A

### Resource availability

#### Lead contact

Further information should be directed to and will be fulfilled by the lead contact Prof. Dr. M. Nieuwdorp (m.nieuwdorp@amsterdamumc.nl).

#### Materials availability

This study did not generate new unique reagents or materials.

### Experimental model and study participant details

#### Study population and ethics approval

This study recruited non-insulin dependent individuals with T2D. Participants were recruited through local newspapers, social network advertisements general practitioners, or during a visit to the center where they were followed-up for their T2D. Inclusion criteria were: presence of T2D as defined by the American Diabetes Association,^60^ stable use of metformin for ≥3 months, as metformin therapy is among the first lines of antidiabetic drugs and has a profound effect on gut microbiota,^61^^,^^62^ BMI≥25 kg/m^2^, age 40–70 years, Caucasian, Caribbean or African origin. Exclusion criteria were: use of insulin therapy, antibiotic usage within the last three months, uncontrolled diabetes (HbA1c>9% i.e., 75 mmol/mol), vegetarian diet, presence of chronic inflammatory disease, use of pre-pro-synbiotics, use of proton-pump-inhibitor, eGFR < 50 mL/min/1.73 m^2^, active malignancy, unmotivated or unable to adhere to the diet. The baseline characteristics of participants are described in Table 1.

The study was approved by the local Institutional Review Board of both centers and was carried out simultaneously in Amsterdam UMC, location AMC and Paris, University Hospital Pitié-Salpêtrière, Sorbonne University. The study was conducted in accordance with the Declaration of Helsinki and registered at the clinical trial registry: NCT03732690 and NCT03732690. Written informed consent was obtained from all participants.

### Method details

#### Study design

This study was a 12-week randomized controlled, non-blinded, isocaloric dietary intervention clinical trial (Figure 1). Participants were randomized to follow either a HP diet (HP) or a LP diet (LP) for a duration of 12 weeks and visited the study location fasted at three times (Week 0, Week 6 and Week 12). Before each visit, participants collected 24 h and fresh fecal samples and delivered them in a cool box. Feces samples were immediately frozen in −80°C.

Two weeks before each study visit, participants wore a continuous glucose monitor (Freestyle Libre) in order to obtain real-life data. At baseline and week 12, participants underwent a MMT to determine insulin resistance, which provides a more physiological response compared to oral-glucose tolerance test.^63^^,^^64^ Primary objective of the study was to investigate the effect of the diet on post-prandial glycemic excursion (AUC) after the MMT. Secondary objectives were to analyze the effects of the diets on (i) post-prandial glycemic excursion using continuous glucose monitors, on glycemic control and metabolic markers such as HbA1c and cholesterol levels, (ii) gut microbiota composition, alpha and beta diversity and (iii) serum gut-derived metabolites levels. All individuals had a moderate to normal renal function, which was defined as an eGFR > 50 mL/min/1.73 m^2^, according to the MDRD formula.^65^^,^^66^

#### Diet

The objective in the HP group was to reach 30% of total energy intake (En%) from protein. The objective in the LP diet (LP) was to limit protein to 10% of total energy intake (En%). These En% were used as target, as they are among the extreme of the reference intake of protein recommended.^67^ In both groups, no caloric restriction was performed. Baseline energy intake requirements were estimated using resting energy expenditure measured by indirect calorimetry adjusted for physical activity level. To avoid the confounding effect of weight changes on the outcomes of this study, participants were instructed to not change their lifestyle throughout this study, with the exception of protein composition in their diet.

Before randomization and during the entire study period, participants had filled out a three-day food diary weekly (two weekdays and one weekend day, randomized every week). Personalized adaptations of their diet were then given by a trained dietician to reach the protein consumption objectives of the allocated group (HP or LP) using a mix of dietary guidance and specific food deliveries to their homes. Participants were provided with HP or LP snacks and meals, which filled approximately half of their total energy intake throughout the study. In the HP group protein supplements consisted mostly out of animal protein, well balanced between red,white meat and fish. The detailed composition of the food supplements is provided in the supplementary information (Table S1) For the other half of their food intake, they were instructed to favor/avoid certain foods using lists of high or LP containing foods and example menus. Throughout the study, participants had weekly phone contact with the dietician to ensure dietary compliance and to provide additional guidance if the protein objectives were not reached. In addition to dietician interviews, compliance to the diet was evaluated using 24-h dietary recalls, collected 3 days per week, so a total of *12∗3=36 food diaries per subject,* and by collecting 24-h urine samples before each study visit in order to determine the urea/creatinine ratio, a validated marker for protein intake.^68^

#### Anthropometric and clinical measurements

At baseline and week 12 (end of study), body composition was measured through Bioelectrical impedance analysis and resting energy expenditure through indirect.^69^ Blood samples were collected after an overnight fast. Fasting serum glucose, triglycerides and HbA1c were measured using enzymatic methods. Examination of these anthropometric and biological outcomes were part of secondary outcomes to be evaluated.

#### Mixed-meal test

Participants underwent a 4-h MMT.^63^ Briefly, participants visited the study center at baseline and week 12. Subjects were fasted for at least 8 h before the site visit and an intravenous catheter was placed in a distal arm vein. Baseline blood sampling was obtained and afterward participants immediately ingested a liquid meal solution (Nutridrink, Nutricia Advanced Medical Nutrition, Amsterdam, the Netherlands) containing 600 kcal (35% fat, 49% carbohydrates and 16% proteins) blood was sampled at fixed time point for the duration of 4 h, centrifuged and stored in the minus 80°C, until further analysis. Blood was drawn for metabolite analyses at baseline (fasted) and after 240 min post-prandial, after ingestion of the MMT. This procedure was done before the dietary protein intervention and after 12 weeks of following either an HP or an LP diet in order to determine (240 min post prandial) plasma metabolite changes modulated via 12 weeks of dietary protein intervention.

#### DNA extraction, library preparation and gut microbiota sequencing analysis

DNA extraction and library preparation was performed as previously published.^70^^,^^71^ Briefly, fecal samples were extracted in Lysing Matrix E tubes (MP Biomedicals) containing ASL buffer (Qiagen). Lysis was obtained after homogenization by vortexing for 2 min followed by two cycles of heating at 90 °C for 10 min with afterward three bursts of bead beating at 5.5 m s-1 for 60 s in a FastPrep-24 instrument (MP Biomedicals). After each bead-beating burst, samples were placed on ice for 5 min. Supernatants containing fecal DNA were collected after two cycles by centrifugation at 4 °C. Supernatants from the two centrifugation steps were pooled, and a 600-μL aliquot from each sample was purified using the QIAamp DNA Mini kit (QIAGEN) in the QIAcube instrument (QIAGEN) using the procedure for human DNA analysis. Samples were eluted in 200 μL of AE buffer (10 mM Tris-Cl, 0.5 mM EDTA, pH 9.0). Libraries for shotgun metagenomic sequencing were prepared by a PCR-free method; library preparation and sequencing were performed at Novogene (Cambridge, UK) on a HiSeq instrument (Illumina) with 150-bp paired-end reads and 6 G data per sample. The MEDUSA pipeline was used to process shotgun metagenomics.^72^ Briefly, Total fecal genomic DNA was extracted from 100 to 150 mg of feces by repeated bead beating using a modification of the IHMS DNA extraction protocol Q.^70^ libraries for shotgun metagenomic sequencing were prepared by a PCR-free method; library preparation and sequencing were performed at Novogene (Cambridge, UK) on an Illumina NovaSeq 6000 S4 flow cell with 150-bp paired-end reads and 6 G data per sample. Raw reads were processed with a pipeline implemented with NGless v.1.0.^73^ Briefly, reads were first preprocessed by filtering basecalls with a *phred* score below 25 and reads with less than 45 bp of length. Then, contaminants were filtered by mapping the quality filtered reads against a database containing human, animal, fungus and plant genomes (minimum match size = 45 and min identity percentage = 95). Filtered reads were mapped with bwa^74^ against the IGC (Integrated Gene Catalog), a 9.9 million human gut microbial genes collection.^75^ Gene abundance table was computed with the NGless *dist1* option where multiple mapped reads are distributed based on the coverage of singly mapped reads. The gene abundance table generated with NGless was then treated with MetaOminer V1.2^76^ for rarefaction to 10^7^ reads and RPKM normalization. A second catalog of Co-Abundance Gene groups (CAG) that accompanied the IGC, those CAGs with more than 500 genes were considered as Metagenomic Species (MGS). The relative abundance for each MGS was established as the average abundance of the 50 most correlated genes. Classification at the species level for each MGS was stablished when at least 50% of the MGS matched the same NCBI reference genome at 95% identity and 90% of the length coverage. For superior taxonomic levels, the criteria were relaxed to 85% and 75% of identity to assign genus and phylum respectively. Finally, abundances of gut microbial derived metabolic modules (GMM) were determined from gene abundance tables according the classification proposed by Vieira-Silva.^77^

#### Metabolomics

Plasma metabolites were measured using stable-isotope-dilution LC-MS/MS as recently described^39^^,^^78^ Quantification of histidine, ImP and urocanate were similarly performed using stable isotope dilution LC-MS/MS, using heavy isotope labeled internal standards (Histidine-D5,15N3, Cambridge Isotope Lab. Inc, ImP-13C3 and urocanate-13C3, Astra Zeneca, Cambridge, UK).^40^

#### Targeted LC-MS/MS analysis of selected metabolites in human plasma

Stable-isotope-dilution LC-MS/MS was used for quantification of metabolites as previously described with some modifications.^78^ An aliquot (20 μL) of plasma was mixed with ice-cold methanolic solution of internal standards (D4-tryptamine; D5-indole-3-acetic acid; D2-indole-3-propionic acid; D5-phenylacetylglutamine; D2-5-OH- indole-3-acetic acid; D5-hippuric acid; 13C2-phenylacetic acid; D4-2-OH-benzoic acid; D4-4-OH-benzoic acid; D7-p-cresol sulfate; D4-indoxyl sulfate; D3-acisoga; D3-L-acetylcarnitine; D7-ADMA; 13C6-arginine; D9-betaine; D9-γ-butyrobetaine; D3-butyrylcarnitine; D3-carnitine; D4-citrulline; D9-choline; D3-creatinine; D9-crotonobetaine; 13C615N-leucine; 13C615N2-lysine; D3-octanoylcarnitine; D6-ornithine; 13C6-phenylalanine; D3-propionylcarnitine; D9-TMAO; D9-trimethyllysine; 13C915N-tyrosine; 13C515N-valine; D4-mannosyl-tryptophan and 13C15N2- pseudouridine) was added to plasma samples (80 μL), followed by vortexing and centrifuging (21,000 × g; 4°C for 15 min). The clear supernatant was then transferred to glass vials with microinserts.

LC-MS/MS analysis was performed on a chromatographic system consisting of two Shimadzu LC-3AD 0 pumps (Nexera X2), a CTO 20AC oven operating at 30°C (for the reverse phase method) and 40°C (for the normal phase method), and a SIL-30 AC-MP autosampler in tandem with 8050 triple quadruple mass spectrometer (Shimadzu Scientific Instruments, Inc., Columbia, MD, USA). The following ion source parameters were applied: nebulizing gas flow, 3 L/min; heating gas flow, 10 L/min; interface temperature, 300°C; desolvation line temperature, 250°C; heat block temperature, 400°C; and drying gas flow, 10 L/min. A tandem of a Luna Silica column (150 mm × 2.0 mm; 5 μm) (Cat # 00f-4274-B0, Phenomenex, Torrance, CA) and a Kinetex C18 column (50 mm × 2.1 mm; 2.6 μm) (Cat # 00B-4462-AN, Phenomenex, Torrance, CA) was used for chromatographic separation under a non-linear gradient of 0.1% propionic acid in water (solvent A) and 0.1% acetic acid in methanol (solvent B). Electrospray ionization in positive mode with multiple reaction monitoring (MRM) was used with the following transitions: m/z 161.00→144.05 for tryptamine; m/z 164.90→148.20 for D4-tryptamine; m/z 176.00→130.10 for indole-3-acetic acid; m/z 181.20→134.25 for D5-indole-3-acetic acid; m/z 190.00→130.10 for indole-3-propionic acid; m/z 191.80→0.05 for D2-indole-3-propionic acid; m/z 206.20→118.10 for indole-3-lactic acid; m/z 265.2→130.15 for PAGln; m/z 270.1→130.15 for D5-PAGln; m/z 192.00→146.10 for 5-OH-indole-3-acetic acid; m/z 194.20→148.10 for D2-5-OH-indole-3- acetic acid; m/z 180.00→105.10 for hippuric acid; m/z 185.00→110.15 for D5-hippuric acid; m/z 195.80→121.10 for 2-OH-, 3-OH- and 4-OH-hippuric acid; m/z 185.00→126.10 for acisoga; m/z 188.10→126.10 for D3-acisoga; m/z 76.10→59.10 for TMAO; m/z 85.00→66.25 for D9-TMAO; m/z 104.00→60.05 for choline; m/z 113.10→69.20 D9-choline; m/z 118.10→58.10 for betaine; 127.00→66.10 for D9-betaine; m/z 162.00→103.00 for carnitine; m/z 165.00→103.05 for D3-carnitine; m/z 146.00→87.05 for γ-butyrobetaine; m/z 155.00→69.20 for D9-γ-butyrobetaine; m/z 144.00→8.10 crotonobetaine; m/z 153.00→66.15 for D9-crotonobetaine; m/z 133.00→116.05 for ornithine; m/z 139.00→76.10 for D6-ornithine; m/z 147.00→130.10 for lysine; m/z 155.00→90.05 for u-lysine; m/z 161.00→84.10 for methyl-lysine; m/z 175.00→84.10 for dimethyl-lysine; m/z 189.00→84.10 for trimethyl-lysine; m/z 198.10→84.10 for D9-trimethyl-lysine; m/z 175.00→60.10 for arginine; m/z 181.00→74.15 for 13C6-arginine; m/z 176.10→70.10 for citruline; m/z 180.00→74.15 for D4-citruline; m/z 189.00→70.05 for monomethyl-arginine; m/z 189.00→84.15 for homoarginine; m/z 190.00→84.05 for homocitruline; m/z 203.00→70.05 for ADMA; m/z 210.00→77.05 for D7-ADMA; m/z 203.00→172.10 for SDMA; m/z 114.10→44.05 for creatinine; m/z 116.90→47.10 for D3-creatinine; m/z 165.90→120.05 for phenylalanine; m/z 172.10→126.05 for 13C6-phenylalanine; m/z 182.10→136.00 for tyrosine; m/z 192.10→145.05 for u-tyrosine; m/z 118.10→55.15 for valine; m/z 124.10→77.05 for u-valine; m/z 132.00→30.20 for leucine; m/z 139.00→92.15 for u-leucine; m/z 132.00→69.00 for isoleucine; m/z 204.00→84.95 for acetyl-carnitine; m/z 207.00→85.00 for D3-acetyl-carnitine; m/z 218.00→84.95 for propionyl-carnitine; m/z 220.90→85.00 for D3-propionyl- carnitine; m/z 232.00→85.00 for butyryl-carnitine; m/z 234.90→85.00 D3-butyryl-carnitine; m/z 367.00→247.00 for mannosyl-tryptophan; m/z 371.00→251.00 for D4-mannosyl-tryptophan; m/z 243.00→153.00 for pseudouridine and m/z 246.00→156.00 for 13C15N2- pseudouridine. Electrospray ionization in negative mode with MRM was used with the following transitions: m/z 135.00→91.00 for phenylacetic acid; m/z 137.00→92.00 for 13C2- phenylacetc acid; m/z 136.9→93.05 for 2-OH-, 3-OH and 4-OH-benzoic acid; m/z 141.50→97.10 for D4-2-OH and D4-4-OH-benzoic acid; m/z 181.10→163.15 for 4-OH- phenyllactic acid; m/z 186.70→107.00 for p-cresol sulfate; m/z 193.90→ 114.10 D7-p-cresol sulfate; m/z 211.90→79.05 for indoxyl sulfate; m/z 215.50→80.00 for D5-indoxyl sulfate and m/z 1730.10 → 93.1 for phenol sulfate.

#### Sample preparation for histidine, Imidazole Propionate and urocanate quantification

For targeted measurement of histidine, ImP and urocanate, plasma samples were precipitated in 1.3 mL glass vials using 3 volumes of ice-cold acetonitrile containing internal standards (Histidine-D515N3, Cambridge Isotope Lab. Inc, ImP-13C3 and urocanate-13C3, Astra Zeneca, Cambridge, UK). After vortexing and centrifugation, the supernatant was transferred to new glass vials and evaporated under a stream of nitrogen. The samples were then reconstituted in 5% HCl (37%) in 1-butanol and placed in oven at 70°C for 1 h allowing the n-butyl ester to be formed. After derivatization, the samples were evaporated and reconstituted in 100 μL of water:acetonitrile [90:10]. The samples were then analyzed using ultra-performance liquid chromatography coupled to tandem mass spectrometry (UPLC-MS/MS). The analytical system consisted of an Acquity UPLC I-class binary pump, sample manager and column manager coupled to a Xevo TQ-XS (Waters, Milford, MA, USA). The samples (2 μL) were injected onto a C18 BEH column (2.1 × 50 mm with 1.7 μm particles, Waters, Milford, MA, USA) and separated using a gradient consisting of water with 0.1% formic acid (A-phase) and acetonitrile with 0.1% formic acid (B-phase). The analytes were detected with MRM using the transitions 212/110 (histidine), 197/81 (ImP) and 195/93 (urocanate).

For the internal standards, the transitions 220/118, 200/82 and 198/95 respectively were used. Calibration curves of histidine, ImP and urocanate were made in methanol and treated the same way as the samples.

### Quantification and statistical analysis

Based on studies and a hypothesized peak-difference in postprandial glucose excursion (13.0 mmol/L in HP group vs. 10.1 LP group; SD 2.8) following the MMT, we calculated that we needed 60 subjects per arm to detect a significant difference in this trial. This number was based on a significance level of 0.05 and 80% power and was calculated using an online power calculation (www.biomath.info/power). Delta changes before and after intervention were calculated and the effect of the intervention (HP vs. LP) on these changes was analyzed using linear mixed effect models generated with lme4 (v1.1.30) and lmerTest (v3.1.3) R packages and linear regression models correcting for baseline value, site center and using subject ID as random effect in the mixed-effect models, to account for baseline differences. For the linear regression model: Delta variable = group +baseline value variable+ center, for the linear mixed effect models delta_change = group+center | ID as random effect. In the metabolic module analysis additional adjustment for ethnicity was added. In addition to determine the significance of the diet in the abundance of the different GMM we compared the fits of the model adjusted for the diet with a simpler model without diet adjustment.

For metabolites related analyses further adjustment was performed on eGFR changes. All statistical analyses were done using the open software program R (version 4.2.1).^79^ FreeStyle Libre data were processed using the CGDA package.^80^ A p value <0.05 was considered statistically significant. Originally, the aim was to include 120 subject per center in order to have enough power to detect ethnic specific effects in sub-group analysis. However, due to the COVID pandemic, the inclusion rate was hampered and the analysis was restricted to the main objective without sub-group analysis.

## Data Availability

• Data reported in this paper will be shared by the lead contact upon reasonable request. • This paper does not report original code. • Any additional information required to reanalyze the data reported in this paper is available from the lead contact upon reasonable request.
