# Supplementary material for: Protein supplementation changes gut microbial diversity and derived metabolites in subjects with type 2 diabetes
Source: iScience. 2023 Jul 25;26(8):107471. doi: 10.1016/j.isci.2023.107471 (PMC10432813; doi:10.1016/j.isci.2023.107471)
Supplement: Document S1. Figures S1–S7 and Table S1 [file mmc1.pdf]

iScience, Volume *26*

Supplemental information

Protein supplementation changes gut microbial diversity and derived metabolites in subjects with type 2 diabetes

Ilias Attaye, Pierre Bel Lassen, Solia Adriouch, Emilie Steinbach, Rafael Patiño-Navarrete, Mark Davids, Rohia Alili, Flavien Jacques, Sara Benzeguir, Eugeni Belda, Ina Nemet, James T. Anderson, Laure Alexandre-Heymann, Arno Greyling, Etienne Larger, Stanley L. Hazen, Sophie L. van Oppenraaij, Valentina Tremaroli, Katharina Beck, Per-Olof Bergh, Fredrik Bäckhed, Suzan P.M. ten Brincke, Hilde Herrema, Albert K. Groen, Sara-Joan Pinto-Sietsma, Karine Clément, and Max Nieuwdorp

Table S1. Detailed food box information related to Figure 1.

FOOD BOXES			LP: low protein	HP: High Protein			
	TYPE OF FOOD	PRODUCT	kcal (per unit)	carb (g/unit)	protein (g/unit)	fat (g/unit)	Quantity per week
**HP DIET**	**HP bead**	HP rye bread	126	3.6	10.5	6.2	3
	**HP snacks**	HP muffin (madeleine) 2/unit	147	18.4	7.8	5.6	4
		HP biscuits (5/unit)	180	14.6	10.8	8.6	2
	**meat/chicken/fish portion**	33g protein portion (4 chicken/4 fish/4 redmeat)	198	3	33	6	12
	**HP ready meal**	HP Ready meal (see detail)	315.1	29.0	21.1	12.9	6
		**Total per day**	**932.1**	**53.9**	**101.1**	**34.6**	
**LP DIET**	**LP bread**	Low GI bread	172	34.8	5.2	0.8	9
	**LP snack**	Low sugar muffin	94	14.1	1.5	4.4	3
	**Fruit snack**	Fruit mix low sugar	56	12.8	0	0	6
	**LP ready meal**	LP ready meal (see detail)	288.9224599	27.39368687	14.71868687	12.37979798	12
		**Total per day**	**938.8**	**126.8**	**38.0**	**28.2**	

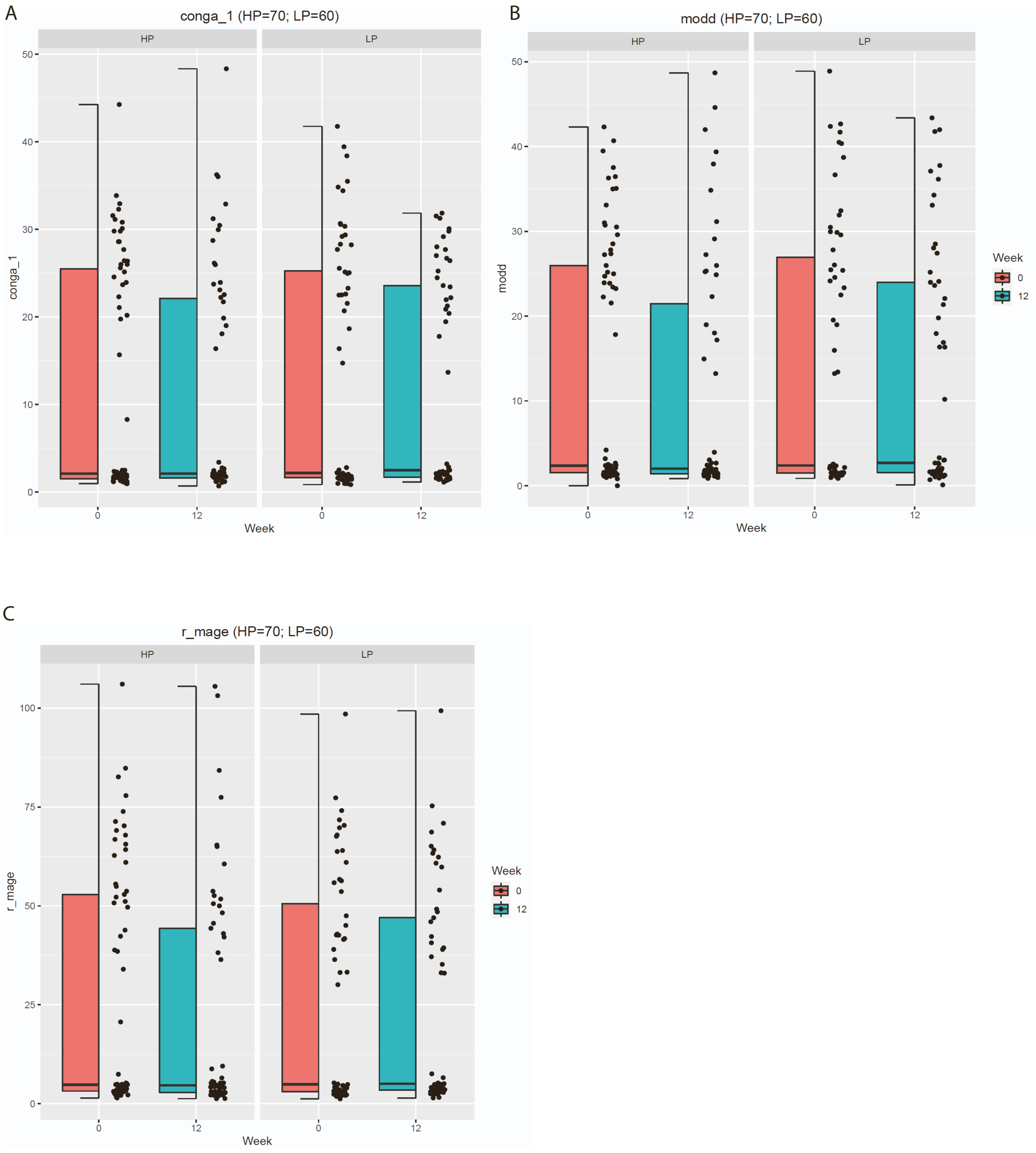

Figure S1. Effect of dietary intervention on glycemic metrics of the continuous glucose monitor, related to Figure 4.

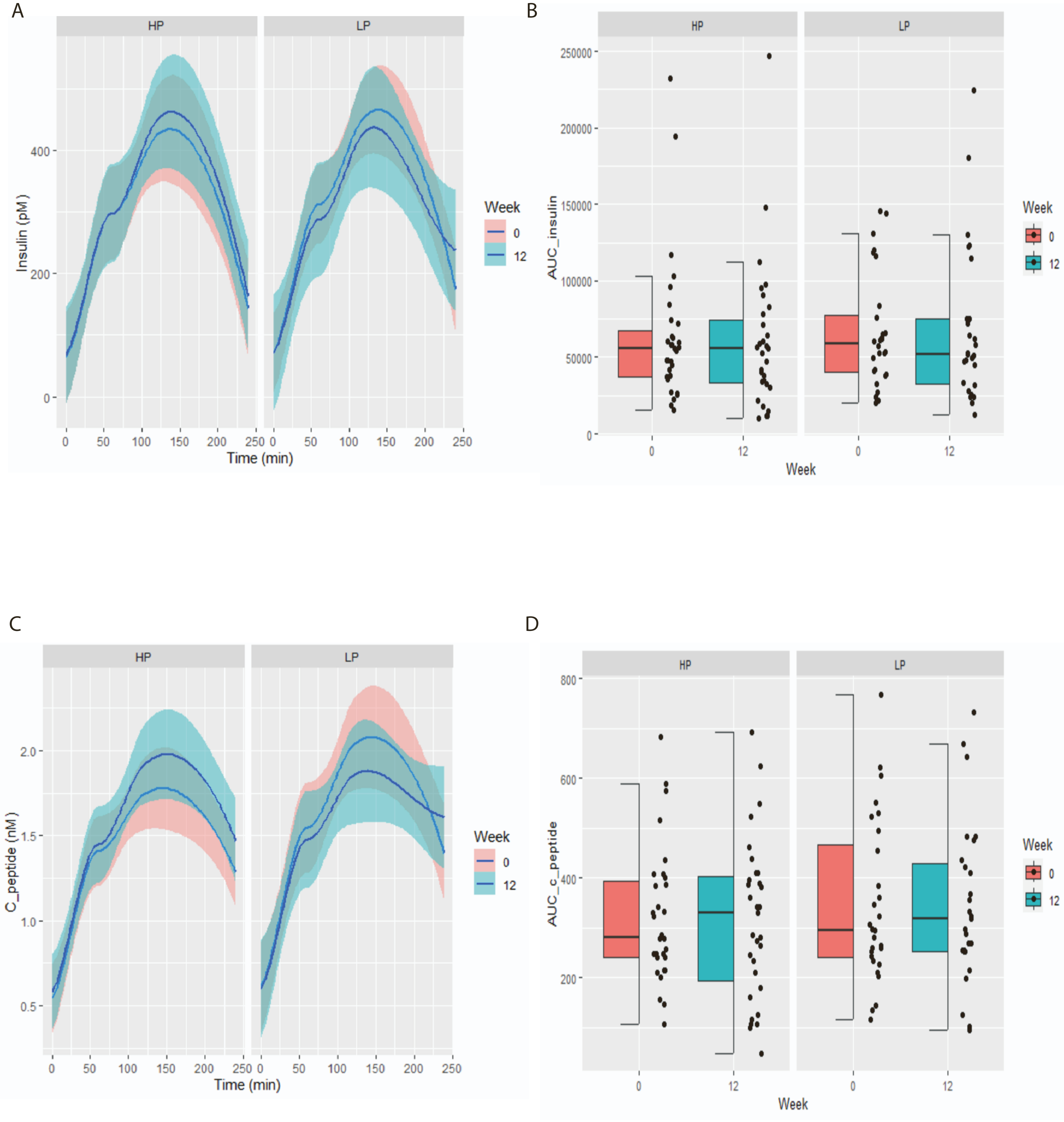

Figure S2. Effect of dietary intervention on (A) Insulin and c-peptide excursions following a mixed-meal test (MMT), related to Figure 4.

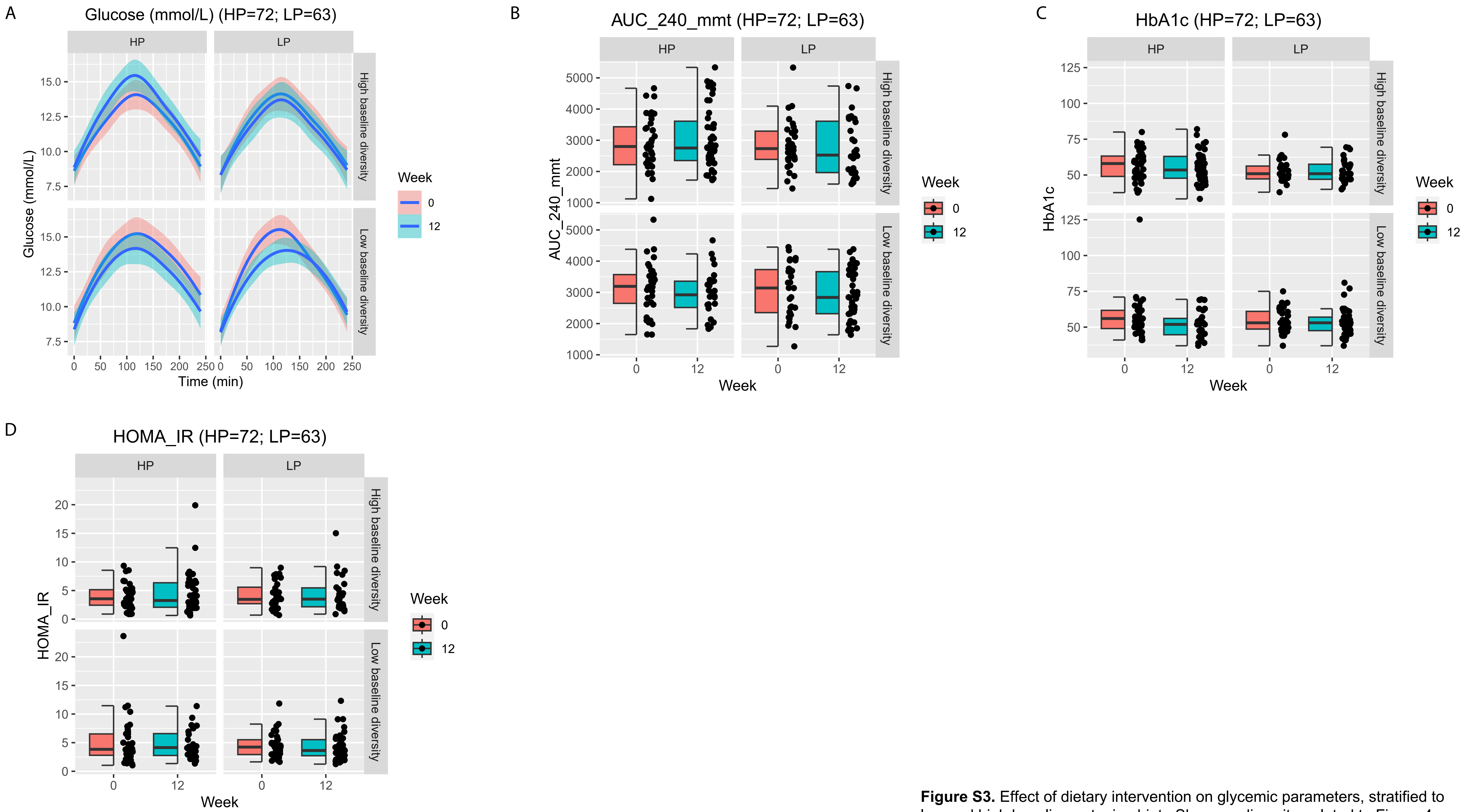

Figure S3. Effect of dietary intervention on glycemic parameters, stratified to low and high baseline gut microbiota Shannon diversity, related to Figure 4.

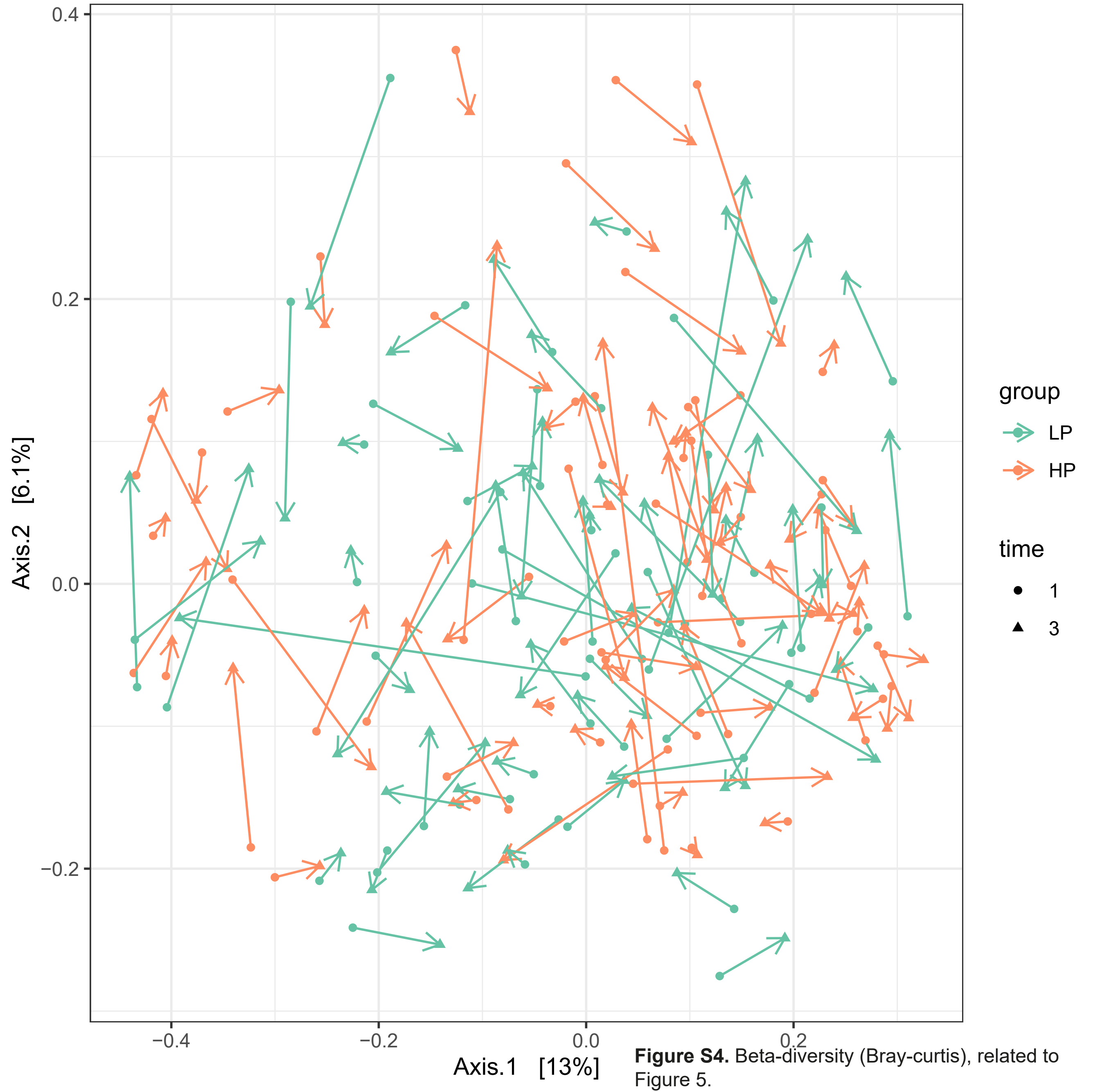

Figure S4. Beta-diversity (Bray-curtis), related to Figure 5.

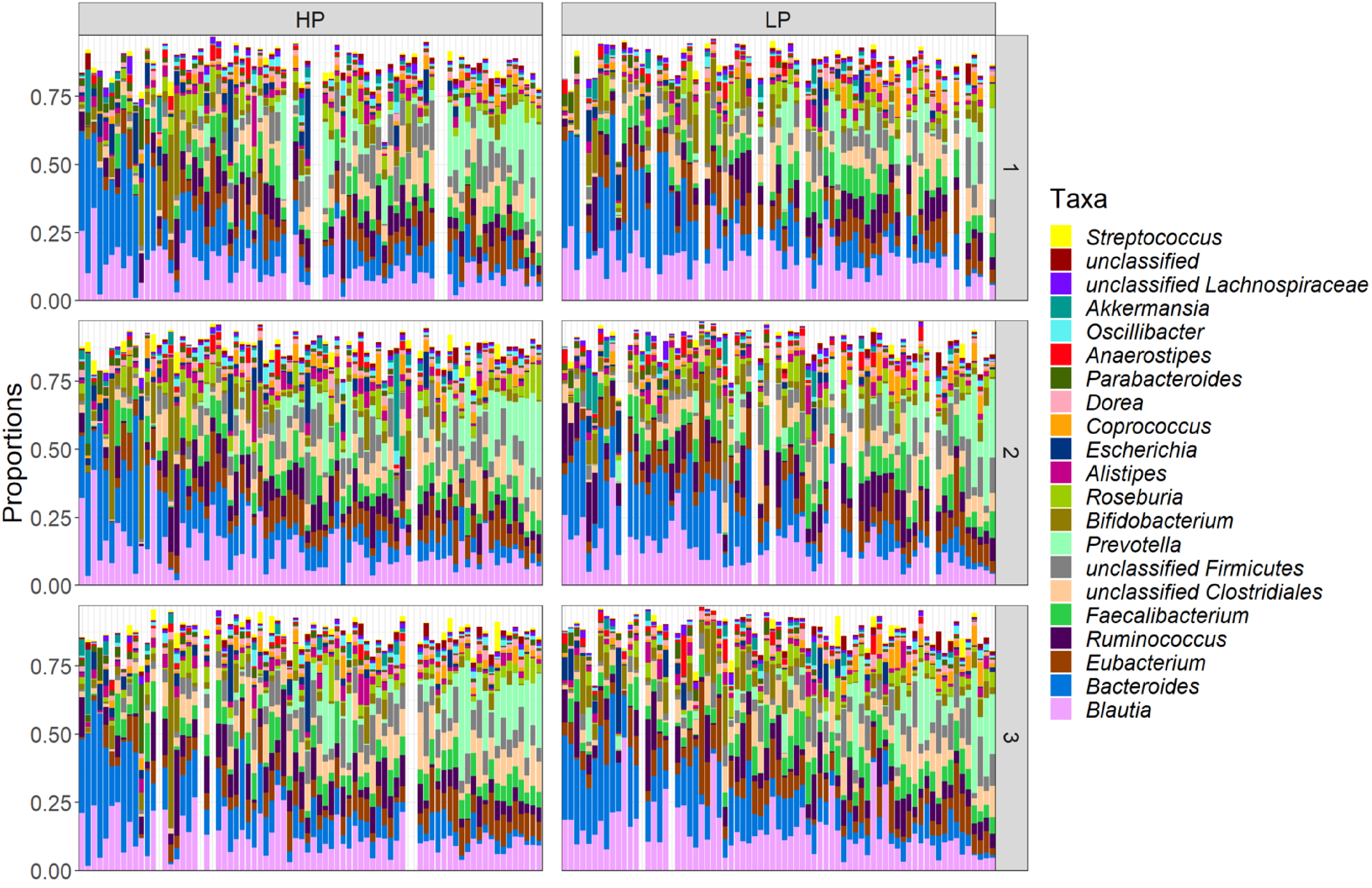

Figure S5. Descriptive Taxonomic changes following a 12 week High protein diet vs Low protein diet, related to Figure 5.

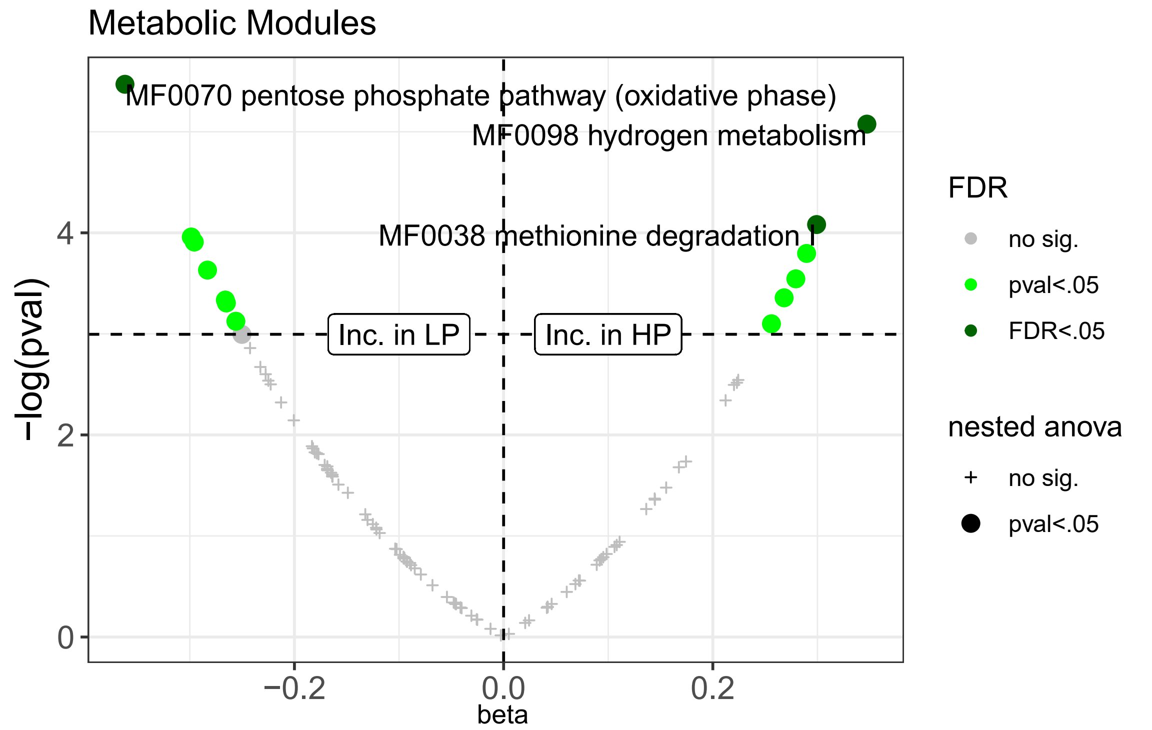

Figure S6. Functional analyses of gut microbiota related to Figure 5.

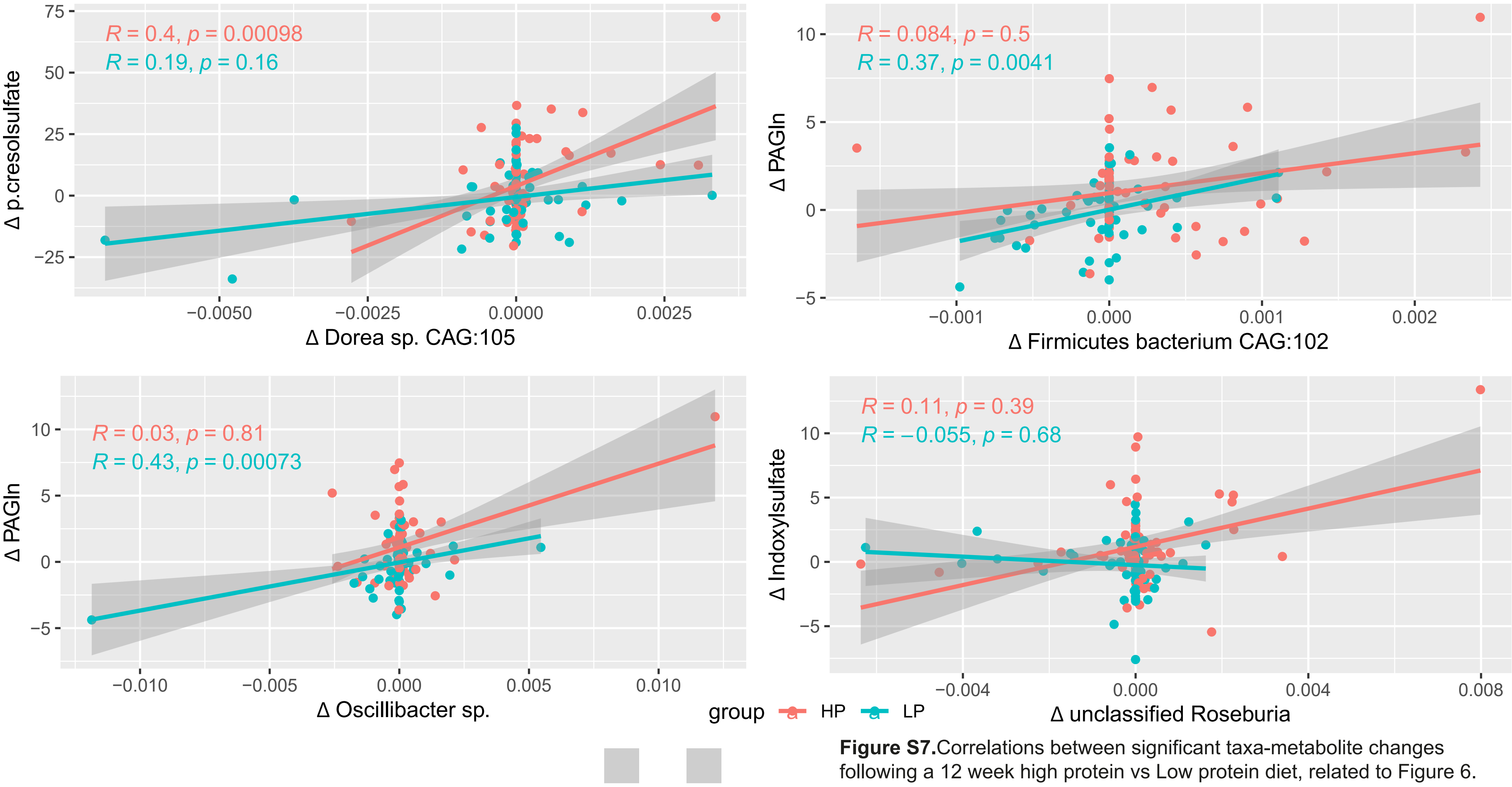

Figure S7. Correlations between significant taxa-metabolite changes following a 12 week high protein vs Low protein diet, related to Figure 6.

Supplemental figure legends

Table S1. Detailed food box information related to Figure 1.

Figure S1: Effect of dietary intervention on glycemic metrics of the continuous glucose monitor. (**A)** Continuous Overall Net Glycemic Action (CONGA). Data represented as median ± IQR, no significant changes between the HP or LP group. **(B)** Mean of Daily Differences (MODD) Data represented as median ± IQR, no significant changes between the HP or LP group. **(C)** Mean Amplitude of Glycemic excursions (MAGE), Data represented as median ± IQR, no significant changes between the HP or LP group. All analyses were performed using linear regression model with correction for baseline value and site center (Delta variable=group +baseline value variable+ center).

Figure S2: Effect of dietary intervention on (**A) I**nsulin excursions following a mixed-meal test (MMT) at Week 0 and Week 12 for 240 minutes. Data represented as mean ± SD, no significant changes between the HP (n=59) or LP group (N=59) . **(B)** Area under the curve (AUC) of post-prandial insulin excursions following MMT-test performed at baseline and Week 12 (ns). Data represented as median ± IQR. (**C)** c-peptide excursions following a mixed-meal test (MMT) at Week 0 and Week 12 for 240 minutes. Data represented as mean ± SD, no significant changes between the HP (n=59) or LP group (N=59). (**D)** AUC of post-prandial c-peptide excursions following MMT-test performed at baseline and Week 12 (ns). Data represented as median ± IQR. All analyses were performed using linear regression model with correction for baseline value and site center (Delta variable=group +baseline value variable+ center).

Figure S3: Effect of dietary intervention on glycemic parameters, stratified to low and high baseline gut microbiota Shannon diversity. (**A)** Glucose excursions following a mixed-meal test (MMT) at Week 0 and Week 12 for 240 minutes. Data represented as mean ± SD, no significant (ns) changes between the HP or LP group, stratified to baseline alpha diversity. **(B)** Area under the curve (AUC) of MMT test performed at baseline and Week 12, stratified to baseline alpha diversity (ns). **(C)** No significant effect of dietary intervention on HbA1c between Week 0 and Week 12, stratified to baseline alpha diversity. (**D)** No significant changes in Homeostatic Model Assessment for Insulin Resistance (HOMA-IR) due to the dietary intervention at baseline and week 12, stratified to baseline

alpha diversity. All analyses were performed using linear regression model with correction for baseline value, baseline microbiota diversity and site center (Delta variable=group +baseline value variable+ center, stratified to high or low baseline microbiota diversity).

Figure S4. Beta-diversity calculated via Bray-curtis index showing an explained variance of the beta-diversity ($p<0.001$). Analyses done via PERMANOVA.

Figure S5. Descriptive Taxonomic changes following a 12 week High protein diet vs Low protein diet.

Figure S6. Functional analyses of gut microbiota showing no significant changes in functional pathways. Analyses performed using a linear mixed effects model.

Figure S7. Correlations between significant taxa-metabolite changes following a 12 week high protein vs Low protein diet. Analyses performed using a linear mixed effects model.
